# Thymosin α1-induced secretion of the IL-15/RA complex by THP-1-derived dendritic cells restrains HIV latency *in vitro*

**DOI:** 10.1080/21505594.2026.2645858

**Published:** 2026-03-13

**Authors:** Chaoyu Chen, Jingna Xun, Jiangrong Wang, Renfang Zhang, Tangkai Qi, Li Liu, Xinyu Zhang, Zichen Song, Yinzhong Shen, Hongzhou Lu, Jun Chen

**Affiliations:** a Department of Infectious Diseases and Immunology, Shanghai Public Health Clinical Center, Fudan University, Shanghai, China; b State Key Laboratory of Genetic Engineering and Engineering Research Center of Gene Technology, Ministry of Education, Institute of Genetics, School of Life Sciences, Fudan University, Shanghai, China; c National Clinical Research Centre for Infectious Diseases, The Third People’s Hospital of Shenzhen, The Second Afliated Hospital of Southern University of Science and Technology, Shenzhen, China

**Keywords:** HIV-1 reservoir, IL-15, thymosin α1, human virtual memory CD8+ T cells, immunological nonresponders

## Abstract

Viral reservoir presents a significant challenge in HIV-1 cure. We previously observed that Thymosin α1 (Tα1) may restrict the reservoir through the IL-15 pathway. However, the precise mechanism remains to be fully elucidated. Peripheral blood mononuclear cells (PBMCs) were obtained from people living with HIV-1 (PLWH). In vitro, THP-1 cells were differentiated into mature monocyte-derived dendritic cells (MoDCs) and co-cultured with PBMCs under various conditions. Intracellular HIV-1 p24 levels, CD8+ T and NK cell functionality, and reservoir size were evaluated. In vitro, Tα1 stimulation of MoDCs resulted in significant immune response and secretion of IL-15/RA complex (*p* < 0.001). This interaction with IL-2 Rβ/γ receptors on T cells enhanced the intracellular secretion of CCL3/5, IFN-γ, and TNF-α in CD8+ T cells (*p* < 0.05), which inhibited p24 levels in CD4+ T cells (*p* = 0.002), and reduced HIV-1 integrated DNA levels (*p* = 0.012). Furthermore, the secretion levels of IFN-γ, TNF-α, and GZMB in NK cells and proportion of CD8+ T_VM_ cells significantly increased following co-culture. These alterations were found to be markedly inversely associated with reservoir size and reactivation. However, these effects were observed in PBMCs from immunological responders (CD4+ T cell count > 350 cells/µL) rather than nonresponders. Tα1 enhances CD8+ T cell function, promotes T_VM_ proliferation, and suppresses reservoir size and reactivation via IL-15 pathway activation in dendritic cells, which warrants testing in functional cure trials in the future.

## GRAPHICAL ABSTRACT

**Figure:**
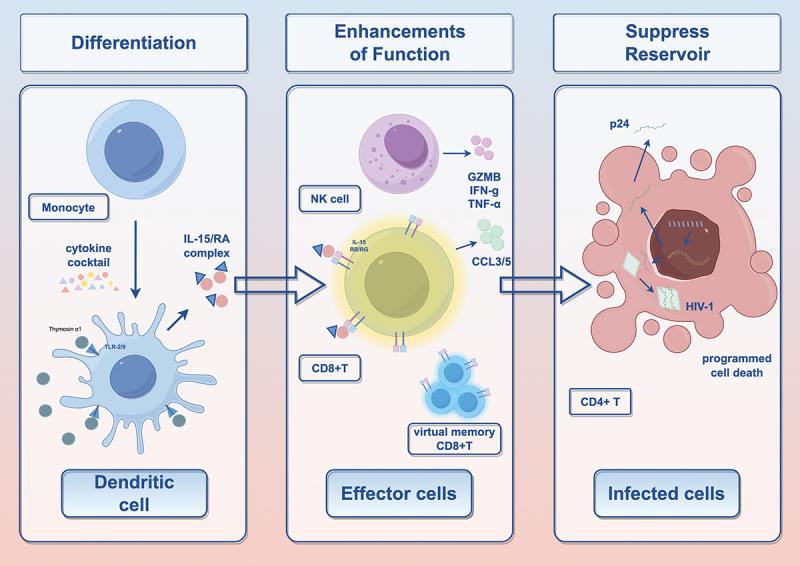

## Introduction

The persistence of HIV-1 reservoirs represents the most significant barrier to achieving a cure for HIV-1 in the post-antiretroviral therapy (ART) era [1]. Current strategies to target these reservoirs involve the use of latency-reversing agents to reactivate latent HIV-1 genomes, followed by a combination of ART and cytotoxic effector cells to diminish the reservoir [2]. In chronic infections, cytotoxic effector cells, including CD8+ T cells and natural killer (NK) cells, play crucial roles in eliminating HIV-1-infected cells. Their robust functionality is closely associated with reservoir control [3]. Effector cells rapidly proliferate upon HIV-1 infection and suppress viral replication and dissemination through both direct cytotoxic mechanisms and non-cytotoxic cytokine/chemokine effects [4]. Compared to healthy individuals, people living with HIV-1 (PLWH) exhibit an increased number of CD8+ T cells but with impaired functionality and an exhausted immune state, which ART alone cannot fully reverse [5]. The functional exhaustion of CD8+ T cells impedes reservoir clearance, potentially linked to inadequate CD4+ T cell recovery.

Recently, the IL-15 superagonist *N*-803 has demonstrated promising effects in individuals with viral suppression. It is considered safe, moderately reactivates HIV-1 provirus, and significantly enhances the cytotoxic functionality of effector cells, effectively reducing the reservoir size in PLWH [6]. Notably, a subset of CD8+ T cells, known as virtual memory CD8+ T cells (T_VM_), has been found to play a unique role in controlling chronic viral infections, including reducing reservoir size in chronic HIV-1 infection [7]. Extensive research in mice has elucidated the differentiation, proliferation, and immunological characteristics of T_VM_, which are highly dependent on IL-15 presented by dendritic cells in the environment [8].

Thymosin α1 (Tα1) is known for its broad immunostimulatory properties and minimal adverse effects and has been used as an adjunctive therapy in acute COVID-19 and chronic hepatitis B [9,10]. Historically, Tα1’s application in HIV-1 has been limited to acute infection stages in combination with antiretroviral drugs, with modest increases in CD4+ T cell counts compared to mono-antiretroviral therapy [11]. Meanwhile, Tα1 enhances the function of various immune cells, including CD8+ T cells [12]. In a previous cohort study, we observed that 24 weeks of Tα1 treatment increased IL-15 levels in a subgroup of PLWH with low CD4+ T cell counts, named immunological nonresponder (INR), and potentially reducing reservoir size [13]. The precise mechanism has not yet been reported. Here, we show that Tα1 stimulates the secretion of the IL-15/IL-15RA complex by monocyte-derived dendritic cells (MoDCs), promotes T_VM_ cell proliferation, improves CD8+ T cell functionality, and reduces reservoir size in peripheral blood mononuclear cells (PBMCs) from PLWH under suppressive ART.

## Methods

### Participants and samples

All participants were enrolled at Shanghai Public Health Clinical Center, Shanghai, China. PLWH who met the following inclusion criteria was recruited: aged 18–75 y, confirmed HIV-1 seropositive, continuous ART > 1 y, and with peripheral HIV-1 RNA levels < 50 copies/mL. Exclusion criteria included active opportunistic infections, pregnancy, drug abuse, non-AIDS related tumors, and severe systemic diseases. INR was defined as PLWH with a CD4+ T cell counts < 350 cells/µL, whereas immunological responder (IR) was above this threshold. PBMCs were isolated from the buffy coat using Ficoll (Stemcell, #18061) density gradient centrifugation in the laboratory, following the manufacturer’s instructions.

### Cells and culture media

The human monocytic leukemia cell line THP-1 was purchased from ATCC (Catalog, #TIB-202). The cells were maintained in tissue culture flasks in a culture medium consisting of RPMI 1640 (Gibco, #22400089) supplemented with 2 mM L-glutamine, 100 IU penicillin, 100 µg/ml streptomycin (Gibco, #15070063), and with 10% (v/v) heat-inactivated fetal bovine serum (FBS; Gibco, # 10099141) in a humidified incubator at 37°C under 5% CO_2_.

### Differentiation of MoDcs

The THP-1 cells were harvested by centrifugation and resuspended in a culture medium without 10% FBS at a concentration of 3x10^5^ cells/ml and transferred in 6-well plates at a final volume of 1 mL per well. To induce the maturation of dendritic cells, a cytokine cocktail was added: rhGM-CSF (100 ng/ml), rhIL-4 (200 ng/ml), rhTNF-α (20 ng/ml), and ionomycin (200 ng/ml) (all from Beyotime, #P5286, #P5129, #P5318, #S1672) [14]. Additionally, a final concentration of 100ug/ml Tα1 (gifted from SciClone Pharmaceuticals Inc., #86978856000058) was added to the experimental group while the control group received blank media. The cells were cultured for 4–6 d and subsequently used for experiments. The half of culture medium was exchanged and supplemented with a double cocktail every 48 h. Upon maturation of MoDCs, the entire culture medium was replaced and non-adherent cells were removed. The same concentrations of rhGM-CSF, rhTNF-α, and ionomycin were added to the medium to maintain the mature phenotype of the MoDCs after differentiation.

### Bulk RNA sequencing

We performed bulk RNA sequencing on mature MoDCs with and without Tα1 stimulation to explore the effect of Tα1 on transcriptional profiling. Sequencing libraries were generated using the rRNA depleted RNA following manufacturer’s recommendations. The libraries were then sequenced on an Illumina Novaseq platform and clean data were obtained by removing reads containing adapters, reads only containing poly-N, and low-quality reads from the raw data. After quality control, paired-end clean reads were aligned to the reference genome downloaded from website using StringTie software. FPKM (expected number of fragments per kilobase of transcript sequence per millions base pairs sequenced) values of mRNAs were calculated in each sample. Genes with a *p* value < 0.05 were assigned as differentially expressed. We plotted a heatmap plot to observe the clustering between the samples and gene ontology (GO) and Kyoto Encyclopedia of Genes and Genomes (KEGG) & Reactome pathway enrichment bubble plots to present the function annotations of top differentially expressed genes. This part of the work was entrusted to Xuran Biotechnology Co., Ltd., Shanghai, China. The raw data have been uploaded to the Sequence Read Archive (SRA) database in fastq format, with the accession number PRJNA1321508.

### Co-culture of MoDCs with PBMCs

We developed an in vitro co-culture model to simulate the overall effect of Tα1 on immune cells in the periphery. This process focuses on the cross-talk of mature MoDCs and PBMCs in the context of the re-activation of HIV-1 reservoir by Vorinostat (SAHA). Briefly, each clinical PBMC sample was split into multiples for self-control at a concentration ratio ranging from 5:1 to 10:1 with MoDCs. PBMCs were seeded in the upper nested compartment of a 6-well transwell plate, with the mature MoDCs seeded on the bottom of the lower plate. The final concentrations of 1 µM SAHA (MCE, #HY-10221) and 10 ng/mL IL-2 (Invitrogen, #PHC0026) were added to both Tα1-treated experimental group and blank control group for 48 h. IL-2 sustained T-cell activity, and SAHA synergized with IL-15 to effectively reactivate HIV-1 reservoir in vitro [15,16]. To ascertain the pivotal role of CD8+ T cells or IL-15 in suppressing the reservoir, additional experimental groups utilized the same co-culture model with CD8+ T cell-depleted PBMCs or IL-15 antibody (R&D, # IC2471P) at a final concentration of 10 ng/mL added to the upper layer.

### Measurement of IL-15/RA, T_VM_ phenotype, and intracellular p24, chemokines/cytokines

ELISA: The cell culture supernatant was collected and centrifuged at 2000 g, and the upper layer was taken to measure the concentration of single IL-15 and IL-15/RA complex with the human IL-15 ELISA Kit (Dakewe, #1111502). For statistical convenience, undetected samples were assigned as concentrations based on the minimum concentration of standard curve (15.6 pg/mL).

Surface staining: The T_VM_ phenotype in humans was defined as positive for anti-CD3 Percp-cy5.5, anti-CD8 APC-H7, anti-CD45RA PE-CF594, anti-KIRpan FITC, and/or anti-NKG2A AF-700 (BD, #552852, #641400, #562298, Miltenyi, #130–117–477, # 130–116–177, #130–114-089) [8,17]. The panel of KIR antibodies included anti-KIR2D, anti-KIR3DL1, and anti-KIR3DL2 (Miltenyi, #130–117–477, # 130–116–177). The phenotypes of NK cells were defined as CD3-CD16+, CD3-CD56+, and CD3-CD16+CD56+ (anti-CD16 PE, anti-CD56 PE-cy7 from Biolegend, #156605, #318317). For surface staining, cells were enriched, stained with Fixable Viability Stain – BV510/BV786 (BD, #564406, #565864) for 30 min at 4°C, and subsequently stained with the aforementioned T_VM_ markers as well as anti-IL2RB BV421 (BD, #565348) for an additional 30 min at 4°C. On the other hand, the phenotype of MoDCs was determined by staining with anti-CD45 FITC, anti-CD14 Percp, anti-CD11c BV510, anti-CD86 PE, and anti-CD83 BV650 (all from Biolegend, #304008, #325614, #301633, #305410, #305242).

Intracellular p24, chemokines/cytokines: For PBMCs, cells were permeabilized using Cytofix/Cytoperm Kit (BD, #554714) after surface staining (plus anti-CD4 BV605, BD, #563865), and then stained with anti-CCL-3 APC, anti-CCL-4 PE-Cy7, anti-CCL-5 PE, anti-IFN-γ BV421, anti-TNF-α PE-CD594 (all from BD, #550498, #561116, #554491, #562040, #562113) for CD8+ T cells, anti-GZMB APC (BD, #556026) for NK cells and anti-p24 FITC (Beckman Coulter, #6604665) for CD4+ T cells. For MoDCs, cells were stained with anti-IL-15 PE and anti-IL-15RA BV650 (both from BD, #554597, #362503) after permeabilization. The cells were fixed in 0.5% formaldehyde and analyzed by a BD LSRFortessa flow cytometer (BD, USA). Data analysis was performed using FlowJo software (Tree Star, USA). Detailed staining panels and gating strategies for all flow cytometry analyses were provided (Figures S1, S2). Specifically, the PBMCs were de-adhesion (Figure S1a), lymphocytes were gated (Figure S1b), and dead cells were removed (Figure S1c). NK cells were selected from CD3- cells (Figure S1d,e) and stained intracellularly (Figure S1f, g, j-m). CD4+ and CD8+ T cells were selected from CD3+ cells (Figure S1d, h, and i for CD8+ T cell-depleted controls), and intracellular staining was performed (Figure S1j-s for CD8+ T cells, t-v for CD4+ T cells). T_VM_ cells and surface staining were followed by live cell selection (Figure S1w-z). The MoDCs also underwent deadhesion and dead cell removal (Figure S2a-c), surface staining (Figure S2d-f), and intracellular staining (Figure S2g-j).

### Relative quantitative PCR

At least 1x10^7^ cells were washed twice in PBS (KeyGen, #KGB5001) and total RNA was extracted using Trizol reagent (Thermo Fisher Scientific, #15596026) as per the manufacturer’s instructions. Quantitative polymerase chain reaction (qPCR) with SYBR (DBI, #DBI-2043) was utilized to compare mRNA expression differences between two groups, with GAPDH (Abmart, #M20006) serving as the endogenous control. The expression levels of mRNA were calculated based on the change in cycling threshold using the 2^−∆Ct^ method. For HIV-1 reservoir quantification, primers were designed for the conserved Alu sequence in the human genome and the LTR sequence of the HIV-1 genome. Two-round nested PCRs were performed to amplify sequences containing Alu sequences and integrated proviral HIV-1 DNA. The first round of PCR amplified sequences containing both Alu sequences and integrated proviral HIV-1 DNA, while the second round specifically amplified sequences of HIV-1 integrated DNA. The Alu-PCR method was used to quantify the amount of HIV-1 integrated DNA in PBMCs under co-culture conditions. The detection of HIV-1 DNA and HIV-1 integrated DNA was entrusted to Supbio Biotechnology Co., Ltd., Guangzhou, China.

## Statistical analysis

The statistical analysis was conducted using GraphPad Prism software version 7.0 (GraphPad Software, USA). The data are presented as either mean ± SD or medians with quartile ranges. The normality of the data was assessed using the K-W test. Comparisons between the two groups were made using either the Student’s t-test or nonparametric Mann-Whitney U test, while paired data were analyzed using either the paired t-test or Wilcoxon’s paired test. Correlations between variables were evaluated using either the Pearson or Spearman correlation test. The level of significance was set at *p*-value < 0.05 for all analyses.

## Results

### Tα1 promotes secretion of IL-15/RA complex in MoDcs

**Figure 1.**
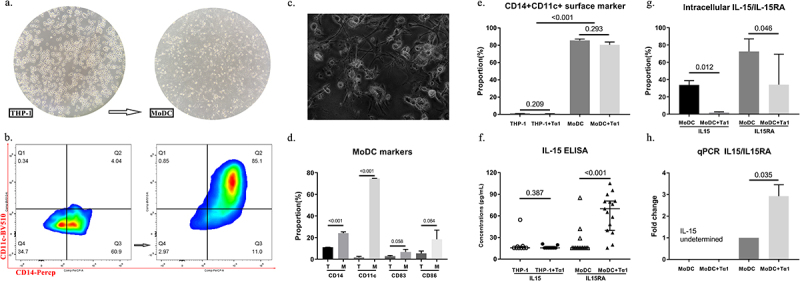
Thymosin α1 promotes secretion of IL-15/RA complex. (a) morphological differentiation from THP-1 to MoDCs after 4-6d (400x). (b) representative gate of mature CD14+CD11c+ MoDCs compared to THP-1. (c) morphological observation of MoDCs under a high-power microscope (1000x). (d) proportions of four surface markers of MoDCs (*n* = 3). (e) proportions of surface marker of THP-1 and MoDCs (*n* = 3). (f) ELISA results of IL-15 and IL-15/RA complex between groups (*n* = 9 for THP-1, *n* = 15 for MoDC). (g) intracellular IL-15 and IL-15RA of MoDCs (*n* = 3). (h) qPCR results of IL15 and IL15RA (*n* = 3). MoDCs: monocyte-derived dendritic cells; qPCR, quantitative polymerase chain reaction. Statistical method used in this figure was unpaired Student’s t-test.

After 4–6 d of differentiation, THP-1 cells underwent a transformation from a spherical, transparent suspended state to mature MoDCs with dense cytoplasm and prominent dendrites (Figure 1a). The proportion of CD14+CD11c+ cells in the surface marker combination used to characterize MoDCs increased from less than 5% to more than 80% (Figure 1b). This indicator clearly distinguishes between MoDCs and THP-1. Morphological features were more conspicuous when noticed under a high-power microscope (Figure 1c). In contrast, immature dendritic cells did not exhibit the aforementioned characteristics (Figure S3a). We measured changes in surface markers in MoDCs compared to THP-1 and observed prominent increases in CD14 and CD11c (*p* < 0.001), while an increasing trend in CD86 and CD83 (*p* > 0.05) (Figure 1d). Importantly, consistent with the results observed in primary CD14+ monocytes [18], Tα1 had no effect on the differentiation of THP-1 to MoDCs (Figure 1e).

The concentration of IL-15 and IL-15/RA complex in cell supernatants was below the lower limit of detection in the majority of samples. However, after Tα1 treatment, the concentration of IL-15 in MoDCs substantially increased (*p* < 0.001) (Figure 1f). We also examined the intracellular levels of IL-15 and IL-15RA in MoDCs. Unexpectedly, both IL-15 and IL-15RA declined after Tα1 treatment (*p* = 0.012; *p* = 0.046) (Figure 1g). Since the detection of IL-15 monomers poses challenges, as they often exist in complex forms, it is reasonable to speculate that Tα1 induces the exocytosis of the IL-15/RA complex, resulting in a reduction in intracellular levels [19]. In addition, at the mRNA level, we observed a significant upregulation of IL15RA but not IL-15 transcripts (*p* = 0.035) (Figure 1h).

**Figure 2.**
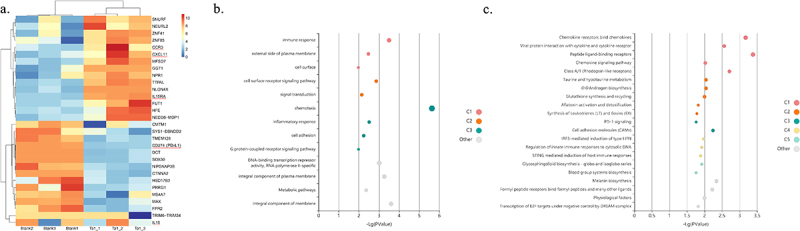
Transcriptional profiling and bioinformatics analysis after Tα1 treatment of MoDCs. (a) heatmap plot of top30 differentially expressed genes. (b) bubble plot of gene ontology enrichment analysis. (c) bubble plot of Kyoto encyclopedia of genes and genomes and Reactome pathway analysis. C1, C2, to others represent the principal component analysis’ result order from high to low proportion of differential genes, which shared between multiple enriched pathways. Statistical method used in this figure was unpaired Student’s t-test.

After coculture with Tα1, a total of 148 genes were found to be differentially expressed in MoDCs (|log_2_FC|>1, *p* < 0.05). The top 30 genes with the lowest *p*-value were selected for further analysis, and the heatmap suggested significant inter-group differences in expression profiles between the blank group and Tα1-treated group, which were clustered into two categories (Figure 2a). In the results of the enrichment analysis, GO analysis highlighted the significance of immune response (*p* < 0.001, Figure 2b), and KEGG & Reactome pathway analysis suggested that chemokine receptors binding chemokines were significantly enriched (*p* < 0.001, Figure 2c). In heatmap, we noticed five genes of interest and found that Tα1 treatment led to the upregulation of CXCL11 and CCR3, both of which play pivotal roles in the activation of T cells. Interestingly, PD-L1 (gene name CD274) expression significantly decreased in MoDCs and the protein encoded by this gene is thought to bind to the PD-1 receptor on T cells to induce T cell functional exhaustion. Our previous cohort study found that the proportions of PD-1 positive T cells in participants significantly decreased after Tα1 treatment [13]. These results collectively indicate that Tα1 can reshape the T cell composition and reverse immune exhaustion to a certain extent. The expressions of the IL15 and IL15RA mRNA were in line with qPCR validation, which is in accordance with our hypothesis that Tα1 promotes extracellular secretion of the IL-15/RA complex rather than transcription.

### Tα1-stimulated MoDCs restrain the viral reservoir through IL-15 pathway

Blood samples from 54 participants, including 23 IRs and 31 INRs, in this study were collected. The overall clinical information is shown in Table 1. The proportion of T_VM_ was significantly higher in IRs than in INRs (Figure S5a) and presented a positive correlation with CD4+ T counts (r2 = 0.312, *p* = 0.014) as well as the CD4/CD8 ratio (r2 = 0.532, *p* < 0.001) (Figure S5b).

**Table 1.** Clinical characteristics of study participants.

	Total (*n* = 54)	IRs (*n* = 23)	INRs (*n* = 31)	*p* value *
Age (year)	44 (32–50)	45 (37–50)	42 (32–51)	0.105
Gender (male/female)	48/6	19/4	29/2	0.206
CD4 count (cells/µl)	267 (133–420)	433 (388–564)	155 (83–235)	<0.001
CD8 count (cells/µl)	523 (368–907)	505 (362–849)	558 (384–955)	0.546
CD4/CD8 ratio	0.40 (0.20–0.90)	0.91 (0.59–1.41)	0.22 (0.11–0.35)	<0.001

Data are shown as median with interquartile except gender.

*Unpaired Student T-test for age, unpaired Mann – Whitney U test for CD4, CD8 count, and CD4/CD8 ratio, and Fisher Chi-square test for gender.

**Figure 3.**
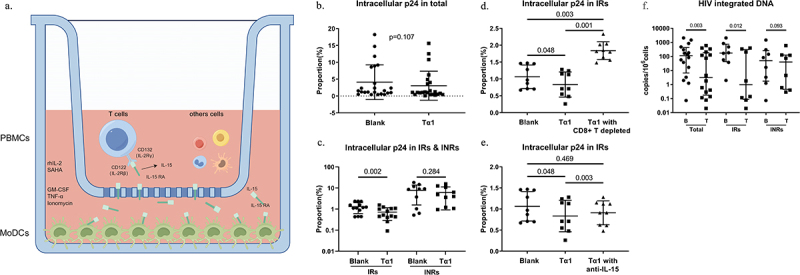
Reduction of HIV-1 reservoir. (a) co-culture model. (b&c) intracellular p24 levels of CD4+ T cells in total and groups (*n* = 23 for total, *n* = 13 for IRs, *n* = 10 for INRs). (d) intracellular p24 levels of CD4+ T cells in CD8+ T cells-depleted PBMCs of IRs (*n* = 9). (e) intracellular p24 levels of CD4+ T cells in PBMCs (including CD8+ T cells) of IRs after IL-15 blockade (*n* = 9). (f) HIV-1 integrated DNA levels of PBMCs in total and groups (*n* = 16 for total, *n* = 8 for IRs & INRs). rhIL-2, recombinant human IL-2; SAHA, Vorinostat; B, blank; T, Tα1. Statistical method used in (b&c) was paired t-test and in (d-f) was Wilcoxon’s paired test.

As illustrated in Figure 3a, the lower layer of the transwell culture plate was seeded with maintained mature MoDCs, which were triggered by Tα1 to secrete IL-15/RA complex and subsequently bind to IL2Rβ/γ receptors (also referred to as IL15Rβ/γ) on PBMCs in the upper layer through small pores. In the co-culture model of MoDCs and PBMCs from these subjects, intracellular p24 showed a decreased trend after Tα1 treatment (*p* = 0.107) (Figure 3b). Notably, a significant decrease in intracellular p24 was observed in IRs (*p* = 0.002, Figure 3c). When CD8+ T cells were depleted from PBMCs using positive magnetic beads, intracellular p24 levels were significantly increased in the IRs (*p* < 0.001, Figure 3d). Moreover, when IL-15 function was blocked with IL-15 antibody in PBMCs, intracellular p24 levels in the IRs showed a rebound increase (Figure 3e). These results suggest that CD8+ T cells play a central role in suppressing the reservoir, especially through the IL-15 pathway. Crucially, HIV-1 integrated DNA, a more intuitive reservoir indicator, was significantly decreased in the total group and IRs (*p* = 0.012), in contrast to the INRs which remained unchanged (Figure 3f).

### Tα1 stimulated MoDC enhanced effector function of CD8+ T cells and NK cells

**Figure 4.**
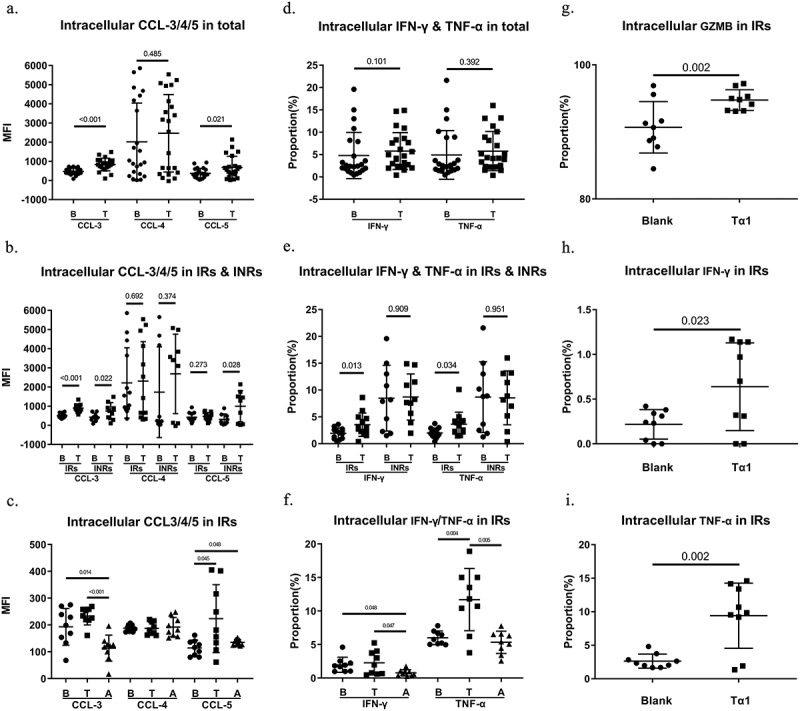
Intracellular cytokines/chemokines of CD8+ T cells and NK cells. (a)&(b) intracellular CCL-3/CCL-4/CCL-5 of CD8+ T cells in total and groups (*n* = 23 for total, *n* = 13 for IRs, *n* = 10 for INRs). (c) intracellular CCL-3/CCL-4/CCL-5 of CD8+ T cells in IRs after IL-15 blockade (*n* = 9). (d)&(e) intracellular IFN-γ/TNF-α of CD8+ T cells in total and groups (*n* = 23 for total, *n* = 13 for IRs, *n* = 10 for INRs). (f) intracellular IFN-γ/TNF-α of CD8+ T cells in IRs after IL-15 blockade (*n* = 9). (g)&(h)&(i) intracellular GZMB/IFN-γ/TNF-α of NK cells in IRs (*n* = 9). B, blank; T, Tα1; A, anti-IL15; MFI, mean fluorescence intensity. Statistical method used in this figure was Wilcoxon’s paired test.

Multiple mechanisms could contribute to this decrease in reactivation levels, including the elimination of latently infected CD4+ T cells. The clinical trials of N-803 have demonstrated that IL-15 primarily enhances the effector function of CD8+ T cells and NK cells, with a relatively modest impact on the reactivation of latently infected CD4+ T cells [6]. We next investigated the role of CD8+ T cells played in the process. We measured intracellular chemokines/cytokines, mainly anti-HIV-1 CCL-3/4/5 as well as broad-spectrum antiviral IFN-γ/TNF-α in CD8+ T cells. It was found that CCL-3 levels were significantly elevated across all subgroups (total: *p* < 0.001, Figure 4a; IRs: *p* < 0.001; INRs: *p* = 0.022, Figure 4b), CCL-4 levels had no trend, and CCL-5 levels were elevated in the total group (*p* = 0.021) as well as INRs (*p* = 0.028, Figure 4a,b). The CD8+ T-cell produced chemokines CCL-3/4/5 have been proven to bind to the HIV-1 co-receptors CXCR4 and CCR5, thereby preventing HIV-1 from entering into CD4+ T cell [20]. The trend of CCL-3 increase in IRs was reversed in the anti-IL15 group (*p* < 0.001, Figure 4c). In addition, IFN-γ and TNF-α were elevated in the IRs (*p* = 0.013; *p* = 0.034) but not INRs or total group (Figure 4d,e), and IL-15 blockade significantly inhibited the increase in IFN-γ and TNF-α levels in IRs (*p* = 0.047; *p* = 0.005, Figure 4f). Additionally, we observed that NK cells in the IRs exhibited increased levels of TNF-α, IFN-γ, and GZMB following Tα1 stimulation (*p* < 0.05, Figure 4g-i). However, due to sample limitations and staining conflicts, blockade experiments were not conducted. These findings indicate that Tα1 enhances the cytotoxic ability of CD8+ T cells (via the IL-15 pathway) and NK cells. Nonetheless, CD8+ T cells in the INR cohort may exhibit functional deficiencies, resulting in a weakened response to beneficial external signals such as IL-15.

**Figure 5.**
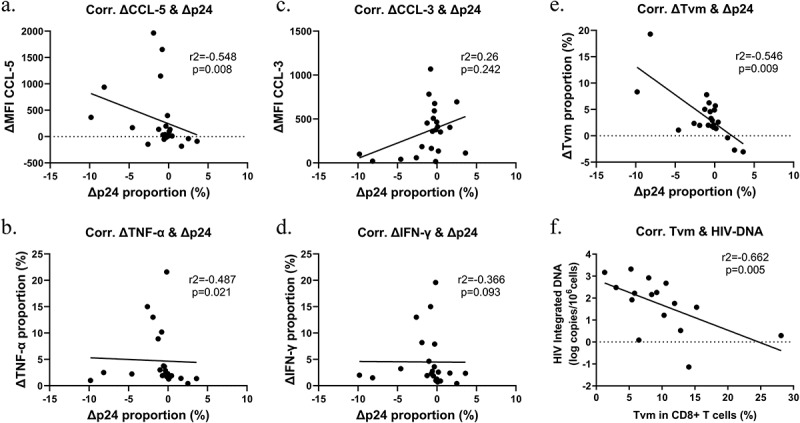
Correlation analysis between indicators of reservoir size and T cells before and after Tα1 treatment. (a) Correlation of ∆MFI CCL-5 with ∆p24 proportion. (b) Correlation of ∆TNF-α proportion with ∆p24 proportion. (c) Correlation of ∆MFI CCL-3 with ∆p24 proportion. (d) Correlation of ∆IFN-γ proportion with ∆p24 proportion. (e) Correlation of ∆T_VM_ proportion with ∆p24 proportion. (f) Correlation of T_VM_ proportion with HIV-DNA copies before Tα1 treatment. ∆, values after treatment subtracts the pre-treated one. T_VM_, virtual memory CD8+ T cells. Statistical method used in (a-e) was Spearman correlation test and in (f) was Pearson correlation test (*n* = 23 for a-e, *n* = 16 for f).

Subsequent evaluation of the dynamic changes between cytotoxic cytokines and p24 after Tα1 treatment revealed a negative correlation between CCL-5 and TNF-α with p24 (CCL-5: r^2^ = −0.548, *p* = 0.008; TNF-α: r^2^ = −0.487, *p* = 0.021; Figure 5a,b). But no correlation was found between CCL-3, CCL-4 (data not shown), and IFN-γ with p24 (Figure 5c,d). Furthermore, the increase in the proportion of T_VM_ after Tα1 treatment was significantly negatively correlated with p24 reactivation (r^2^ = −0.546, *p* = 0.009; Figure 5e), suggesting that the T_VM_ subset may be key cells that inhibit reservoir reactivation. Due to the limited number of cells after co-culture, there was insufficient data of post-treatment HIV-DNA, while a negative correlation was observed between the baseline T_VM_ proportion and HIV-DNA before Tα1 treatment (r^2^ = −0.662, *p* = 0.005; Figure 5f), indicating that a higher T_VM_ proportion was associated with a smaller reservoir size.

To figure out whether the very low percentage of dendritic cells in PBMCs are involved in the IL-15 signaling pathway under co-culture conditions, we additionally measured their ability to secrete IL-15/RA complex in several samples. The results indicated that Tα1 did not have a significant impact on the proportion of CD14+CD11c+ DC in PBMCs (Figure S3b), as well as the proportions of intracellular IL-15/RA complex in both DC cells and total lymphocytes of PBMCs (Figure S3c). Similarly, we investigated the impact of SAHA on the ability of mature MoDCs to secrete IL-15/RA complex and did not observe any impairment in function (Figure S3d). In addition, a differentiation process was undertaken from THP-1 to macrophage precursor M0 cells with CD14+CD11b+ surface markers (Figure S3e). The differentiation efficiency was too low (less than 15%), and Tα1 had no effect on the differentiation process (Figure S3f). However, Tα1 treatment also promoted the secretion of IL-15 by M0 cells (Figure S3g), indicating that macrophage may have similar potential to activate the IL-15 pathway. In addition, to rule out the potential confounding effects of chemokines/cytokines cocktails utilized to maintain mature MoDCs on T cells in co-culture model and to confirm the integral role of MoDCs in Tα1 function, we devised and assessed the impact of culture conditions on T cell function in the absence of MoDCs. In the context of activation of the reservoir by SAHA, neither the chemokines/cytokines cocktail including GM-CSF, TNF-α, Ionomycin, and IL-2, nor single Tα1, nor both agents concomitantly, had a discernible impact on reactivation levels (Figure S4a) and effector function of CD8+ T cells (Figure S4b,c) compared to the blank control.

### Tα1 induces proliferation of T_VM_ and enhances IL2RB expression

**Figure 6.**
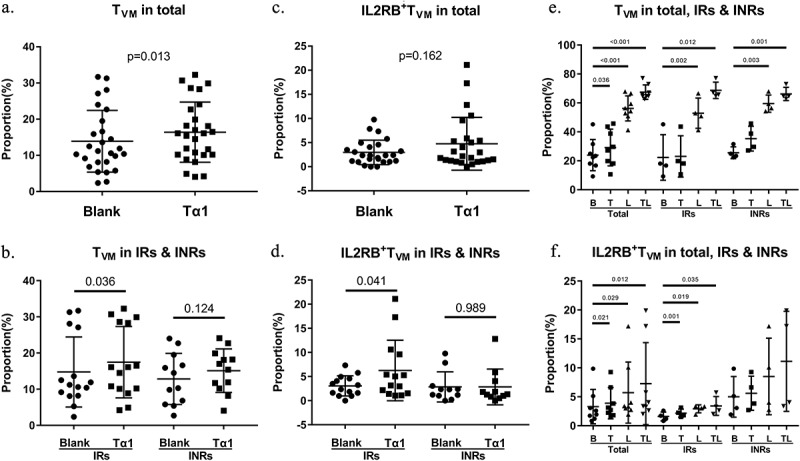
Thymosin α1 induces proliferation of T_VM_ in co-culture model. (a)&(b) proportions of T_VM_ in total and groups. (c)&(d) IL2RB expression of T_VM_ in total and groups. (e) proportions of T_VM_ in total and groups after Tα1 and IL-15 stimuli. (f) IL2RB expression of T_VM_ in total and groups after Tα1 and IL-15 stimuli. B, blank group; T, Tα1; L, recombinant human IL-15; TL, Tα1 plus recombinant human IL-15. Statistical method used in (a-d) was paired t-test and in (e&f) was Wilcoxon’s paired test (in a-d, *n* = 28 for total, *n* = 16 for IRs, *n* = 12 for INRs; in e-f, *n* = 8 for total, *n* = 4 for IRs & INRs).

Previous studies have identified the crucial role of T_VM_ in recognizing and killing reactivated infected cells, thus restraining reservoir size [7]. Considering that the differentiation and proliferation of T_VM_ are dependent on IL-15 signaling presented by dendritic cells, we suggest that Tα1-induced secretion of IL-15/RA complex by MoDCs facilitates the proliferation of T_VM_ sub-population in PBMCs. Indeed, the proportions of T_VM_ among CD8+ T cells significantly increased after Tα1 treatment in total group (*p* = 0.013) and IRs (*p* = 0.036) (Figure 6a,b). T cells presented up-regulated expression of the IL2Rβ receptor upon stimulation with IL-2 and IL-15 [21]. We found that the proportions of surface IL2Rβ receptors on T_VM_ were elevated in IRs after Tα1 treatment (*p* = 0.041), and a similar trend with no significance was observed in the total group, while no change was detected in INRs (Figure 6c,d). A plausible hypothesis is that the T_VM_ in INRs have impaired responsiveness to IL-15 stimulation and thus fails to achieve homeostatic proliferation. To investigate further, we stimulated PBMCs from participants with Tα1 and 20 ng/mL recombinant human IL-15 in vitro and assessed T_VM_ proportions and IL2Rβ expression. As shown in Figure 6e, the usage of IL-15 alone or the combined administration of Tα1 and IL-15 substantially promoted T_VM_ proliferation in the total (Tα1, *p* = 0.036; IL-15, *p* < 0.001; combined, *p* < 0.001) and sub-groups (IL-15 and combined, *p* < 0.05, in both IRs and INRs). Interestingly, single or combined usage of IL-15 and Tα1 increased IL2RB expression in total and IRs, but not in INRs (*p* < 0.05, in both IRs and total, Figure 6f). These findings suggest that IL-15 produced by Tα1 stimulation can promote T_VM_ proliferation by up-regulating IL2RB expression, thereby exerting antiviral effects. Despite the impaired IL2RB expression, the T_VM_ proportion in INRs remained significantly elevated probably because of the high concentration of IL-15, which compensated for the deficiency in IL2RB expression.

## Discussion

The primary obstacle in curing HIV-1 is the reservoir, with even larger reservoir observed in INRs [22]. Currently, CD8+ T cells and NK cells are regarded to play a vital role in controlling chronic HIV-1 infection [23,24]. The classical “shock and kill” strategy aims to eliminate reactivated, latently infected CD4+ T cells, reducing the reservoir to a size where integrated HIV-1 proviruses can no longer harm the host, even in the absence of ART [2]. However, traditional latency reversal agents face two limitations: an inability to effectively reactivate latent cells and impairments of the function of effector T cells and NK cells [25,26]. Remarkably, IL-15 has demonstrated effectiveness in both reactivating the reservoir and enhancing the function of CD8+ T cells and NK cells, thereby achieving dual benefits [6]. Additionally, mature dendritic cells have been shown to induce non-cytotoxic anti-HIV-1 responses in CD8+ T cells through the IL-15 signaling pathway [27]. In this study, we have discovered that Tα1 not only enhances IL-15 signaling in MoDCs and promotes T_VM_ proliferation but also enhances the function of CD8+ T and NK cells and restrains HIV-1 reservoir in vitro.

Tα1 has been proven to regulate many important genes involved in cellular metabolism and immune response in human PBMCs. Specifically, it up-regulates mRNA and protein expression levels of genes including MHC class I, IFN-γ, and MAP-Kinase cascade enzymes. It also enhances the transcription of immune response and chemokines/cytokines related genes such as IL-7, IL-18, IL15R, KLRC, GZMA, MCP-3, RANTES (CCL-5), and SDF-1 [28]. Importantly, after co-culturing with Tα1 for 48 hours, PBMCs from healthy individuals exhibit resistance to HIV-1 and HTLV-1 infection, while CD8+ T cells from PLWH show increased expression of various anti-HIV-1 genes including MIP-1a (CCL-3), MIP-1b (CCL-4), and RANTES [29]. These findings prompt a further exploration of the underlying mechanisms by which Tα1 influences CD8+ T and NK cell function and HIV-1 replication.

In our previous cohort study involving INRs, we observed that treatment with Tα1 for 24 weeks led to an overall increase trend in CD4+ T cell counts and a decrease in the proportion of peripheral T cells expressing PD-1 [13]. Interestingly, we found Tα1 treatment significantly downregulated PD-L1 (CD274) expression in MoDCs (Figure 2a), suggesting that Tα1 may enhance CD8+ T cell function through a dual mechanism: providing activating signals via promoting IL-15/RA complex secretion on the one hand and reducing inhibitory signals via PD-L1 downregulation on the other hand. Future studies are warranted to further elucidate the relative contributions of the IL-15 pathway and the PD-L1 pathway in Tα1-mediated immune reconstitution. In a paired analysis of three cohort samples, we observed a decreasing trend in HIV-1 DNA levels in INRs treated with Tα1 (Figure S6a). Additionally, we noted an increase in IL-15 levels as well as elevated levels of CCL-3/4/5 (Figure S6b-e). It is important to note that due to the limited size of the samples retained, these results did not reach statistical significance (*p* > 0.05). IL-15 and sIL-15 play important roles in promoting CD8+ T cell function and have been implicated in anti-tumor and anti-infectious disease responses [30,31]. Interestingly, the transcription and secretion of IL-15 are not synchronized, and in the absence of IL-15RA involvement, IL-15 is rarely transported to the cell surface or secreted into the extracellular space [19]. Our findings suggest that Tα1 enhances IL-15RA expression and facilitates the extracellular secretion of the IL-15/RA complex. It is important to note that the observed inhibitory effect on the reservoir in this study is restricted to IRs rather than INRs. This may be attributed to the lack of normal responsiveness of CD8+ T cells, particularly T_VM_ cells, to IL-15 pathway in INRs.

Previous studies have demonstrated that mature dendritic cells can enhance non-cytotoxic anti-HIV-1 responses in CD8+ T cells through IL-15 signaling, highlighting the importance of dendritic cells between IL-15 and CD8+ T cell functionality [27]. Tα1, through TLR2/9 stimulation, induces a Th1 type response and IL-12 secretion in dendritic cells, but there is no reporting on IL-15 [32]. Interestingly, the differentiation and proliferation of the T_VM_ subset rely on IL-15 signaling from dendritic cells, and T_VM_ cells exhibit excellent bystander killing capacity [8,33,34]. An important study reported the ability of T_VM_ cells to recognize and kill reactivated infected cells, where IL-15-stimulated HIV-1-specific T_VM_ cells demonstrate stronger cytotoxicity compared to conventional CD8+ T cells [7]. Furthermore, another research found a negative correlation between the secretion of CCL-5 by T_VM_ cells and reservoir levels [35]. These two studies corroborate our findings, providing further evidence for T_VM_ involvement in suppressing the HIV-1 reservoir by killing reactivated infected cells. Our research introduces a novel perspective, suggesting that Tα1 can enhance the dialogue between dendritic cells and T cells via the IL-15 pathway. This interaction promotes the proliferation of T_VM_ and augments the cytotoxic capacity of CD8+ T cell, thereby restraining the HIV-1 reservoir. The subsequent value of this study needs to be confirmed by further phase II clinical trials, which is one of the objectives we intend to pursue in the future research.

Our study has some limitations. First, the sample size of the study is relatively small, which may overlook some valuable discoveries. Second, the use of THP-1 cells instead of primary monocytes as the precursor cells for dendritic cells may limit the comparability with similar studies. We must acknowledge that the THP-1 differentiation system employed in this study exhibits inherent cellular heterogeneity. The culture may contain a mixture of undifferentiated THP-1 cells, immature DCs, and mature DCs. Even with optimized THP-1 differentiation protocols, CD83 expression in mature DCs was only approximately 35%, making it challenging to obtain highly purified single-cell populations [36]. However, the core objective of this study was to validate whether Tα1 could activate the IL-15 pathway and restrain reservoir, our functional data are sufficient to support core conclusions [37].

## Conclusions

Tα1 demonstrated the capacity to activate the IL-15 pathway, augment the function of CD8+ T cells, foster the proliferation of T_VM_ cells, and constrain HIV-1 replication and reservoir size. This marks the inaugural identification of Tα1’s potential to restrain the HIV-1 reservoir, thereby offering a new therapeutic avenue toward achieving eradication of the reservoir.

## Supplementary Material

Supplemental Material

## Data Availability

The raw data supporting the conclusions of this study are available on public to all investigators. These data do not contain any personal information about the participants. Raw data were stored at the Science Data Bank under the CC BY-NC-SA 4.0 protocol. The dataset is accessible at: https://doi.org/10.57760/sciencedb.23008.
