# Supplementary material for: Thymosin α1-induced secretion of the IL-15/RA complex by THP-1-derived dendritic cells restrains HIV latency in vitro
Source: Virulence. 2026 Mar 13;17(1):2645858. doi: 10.1080/21505594.2026.2645858 (PMC13011586; doi:10.1080/21505594.2026.2645858)

**Appendices**

**Figure S1: FACS gating analysis of NK cells and T cells including T_VM_ cells.** (a) singlets. Signals from adherent multicellular aggregates were excluded, and the population of single cells were retained by combining the FSC-H and FSC-A parameters. (b) lymphocytes. Appropriate lymphocyte population were selected based on experimental experience by combining the FSC-A and SSC-A parameters. (c) live/dead cells. Through Fixable Viability Stain BV510 staining, the stained dead cells are excluded. (d) CD3- cells and CD3+ T cells. (e) CD3-CD16+CD56+ NK cells. (f&g) GZMB+ NK cells and FMO negative control. (h) CD3+CD4+ T cells and CD3+CD8+ T cells. (i) CD8+ T cells depleted control. (j&k) IFN-γ+ NK/ CD8+ T cells and FMO negative control. (l& m) TNF-α+ NK/ CD8+ T cells and FMO negative control. (n&o) CCL-3+ CD8+ T cells and FMO negative control. (p&q) CCL-4+ CD8+ T cells and FMO negative control. (r&s) CCL-5+ CD8+ T cells and FMO negative control. (t) CD4+ T cells unstimulated control. (u&v) p24+CD4+ T cells and FMO negative control. (w) CD3+CD8+ T cells. (x) CD3+CD8+CD45RA+ T cells. (y) CD3+CD8+CD45RA+NKG2A+ and/or KIR-pan+ T_VM_ cells. (z) IL2RB+ T_VM_ cells. FSC-H, Forward Scatter-Height; FSC-A, Forward Scatter-Area; FSC-A, Forward Scatter-Area; SSC-A, Side Scatter-Area; FMO, Fluorescence Minus One. Multiple distinct staining panels were applied through the reuse of limited staining channels.

*The flow cytometer we used could stably run staining for a maximum of 10-12 channels (the remaining channels were unavailable or yield unstable results). Therefore, there is channel overlap between multiple different staining protocols. The green box represents the NK cell staining protocol, the red box represents the T cell staining protocol, and the orange box represents the T_VM_ cell staining protocol. Only the intracellular staining protocol provides FMO negative controls.


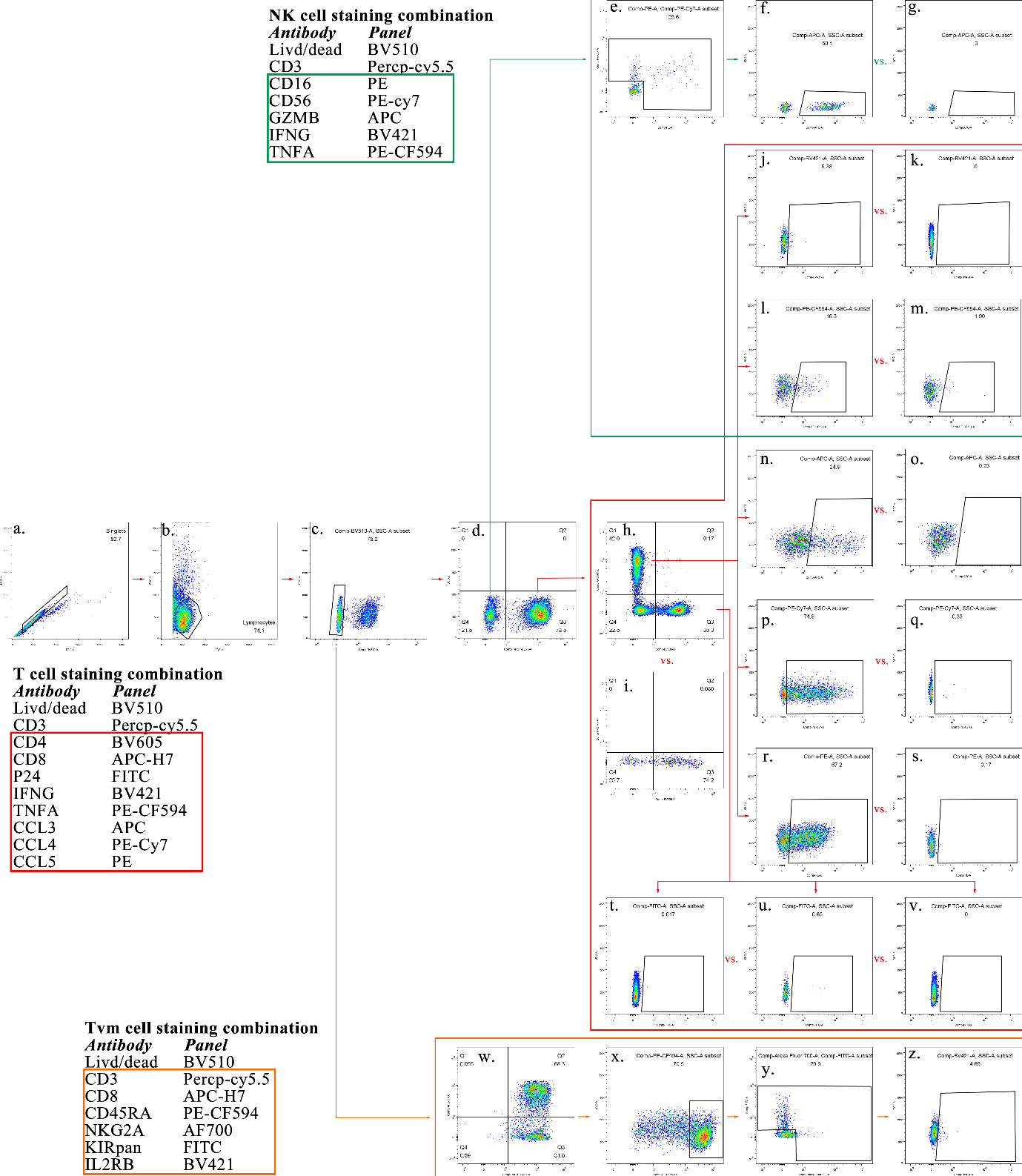


**Figure S2: FACS gating analysis of MoDCs.** (a) singlets. (b) myeloid cells. Appropriate myeloid cell population including theoretic monocyte/macrophage/dendritic cells were selected based on experimental experience by combining the FSC-A and SSC-A parameters. (c) live/dead cells. Through Fixable Viability Stain BV786 staining, the stained dead cells are excluded. (d) CD45+ cells. (e) CD45+CD14+CD11c+ MoDCs. (f) THP-1 undifferentiated control. (g&h) IL-15+ MoDCs and FMO negative control. (i&j) IL-15RA+ MoDCs and FMO negative control. FSC-A, Forward Scatter-Area; SSC-A, Side Scatter-Area; FMO, Fluorescence Minus One.


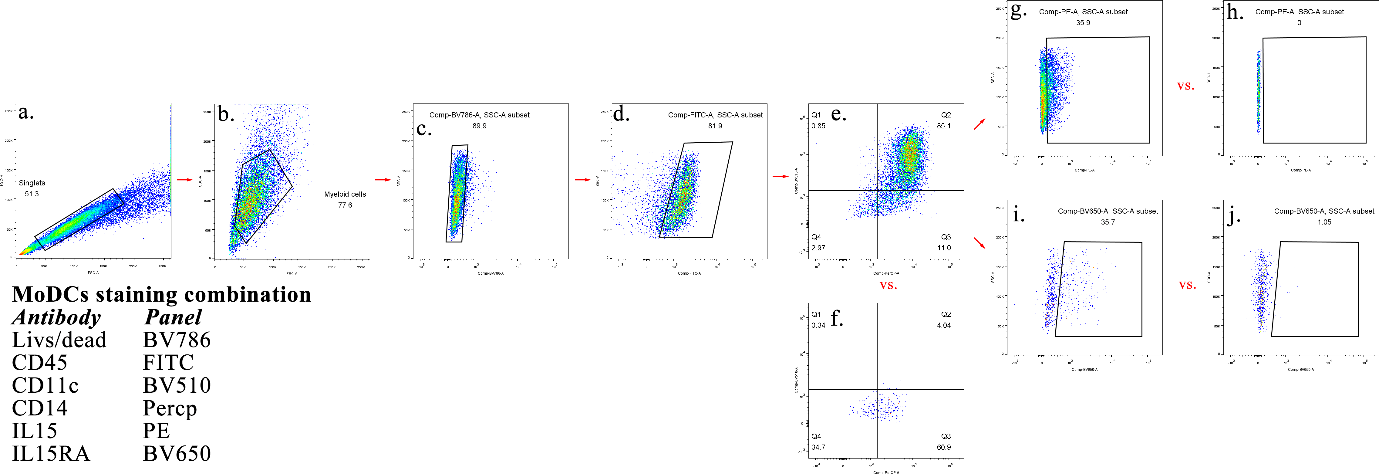


**Figure S3: Dendritic cells in PBMCs and SAHA had no role in IL-15 pathway under co-culture conditions (related to Figure 1).** (a) morphological differentiation from THP-1 to immature DC after 4-6d (400x). (b) proportions of CD14+CD11c+ DC in PBMCs (n=3). (c) IL-15/RA complex in total lymphocytes and DC subsets (n=3). (d) concentration of IL-15 and IL-15/RA complex after SAHA treatment (n=9). (e) morphological differentiation from THP-1 to M0 cells (400x). (f) proportions of surface marker of M0 cells (n=3). (g) concentration of IL-15 and IL-15/RA complex between M0 groups (n=6). M0, precursor macrophage; DC, dendritic cells. Statistical method used in (b&c&f) was unpaired Student's t-test and in (d&g) was unpaired Mann-Whitney U test.

* The method from THP-1 differentiation to immature DCs was roughly the same as differentiation to MoDCs (like culture medium, cell concentration, incubation time), except that only rhGM-CSF (100 ng/ml), rhIL-4 (200 ng/ml) were added to the medium, without rhTNF-a and ionomycin. In addition, the condition for differentiation from THP-1 to M0 cells was to add 100 ng/ml of PMA to the same medium and incubate for 1-2 days.


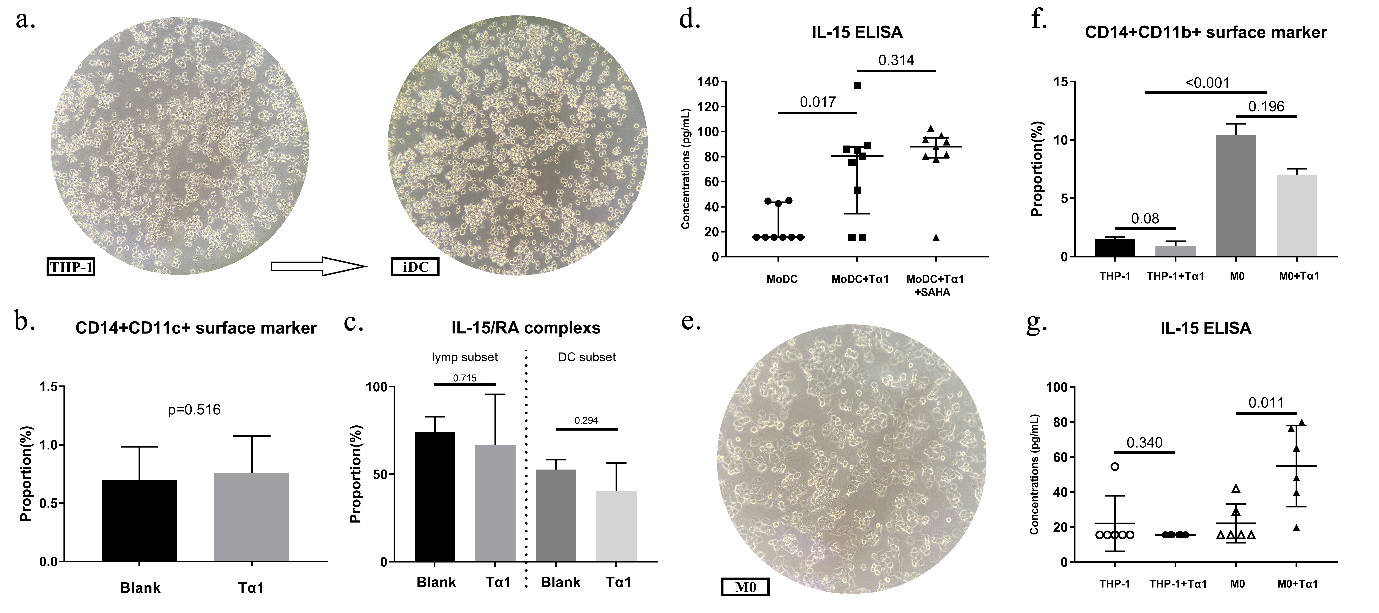


**Figure S4:** **Chemokines/cytokines cocktail and Tα1 had no effects on T cells without MoDCs (related to Figure 3 & Figure 4a-f).** (a) intracellular p24 levels of CD4+ T cells in four groups (n=11). (b) intracellular CCL-3/CCL-4/CCL-5 levels of CD8+ T cells in four groups (n=12). (c) intracellular IFN-γ/TNF-α levels of CD8+ T cells in four groups (n=12). There was no statistical difference between any two groups for any of the indicators. S, SAHA; C, chemokines/cytokines cocktail; T, Tα1. Statistical method used in this figure was Wilcoxon's paired test.

* The samples were derived from PBMCs of IRs, which were directly divided into 4 groups spread to the bottom of the six-well plate, and the results were detected after 48h of incubation in complete medium with the chemokines/cytokines cocktail and Tα1 mentioned above.


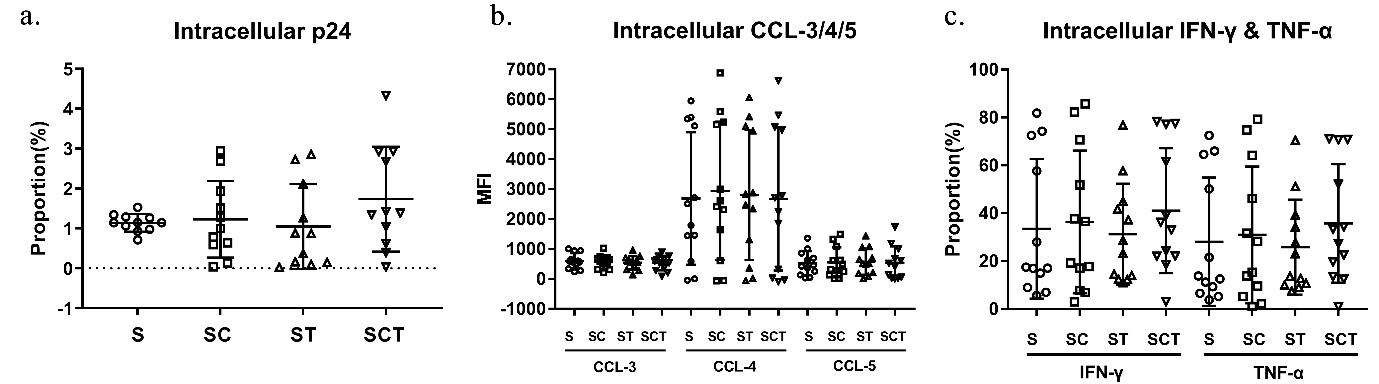


**Figure S5: Phenotype of T_VM_ in people living with HIV-1 (related to Figure 5e&f).** (a) proportions of T_VM_ between INRs and IRs (b) correlation analysis of proportions of T_VM_ with CD4 and CD4/CD8 ratio. T_VM_, human virtual memory CD8+ T cells. INRs, immunological nonresponders; IRs, immunological responders. Statistical method used in (a) was unpaired Student's t-test and in (b&c) was Pearson correlation test (n=54 for total, n=23 for IRs, n=31 for INRs).


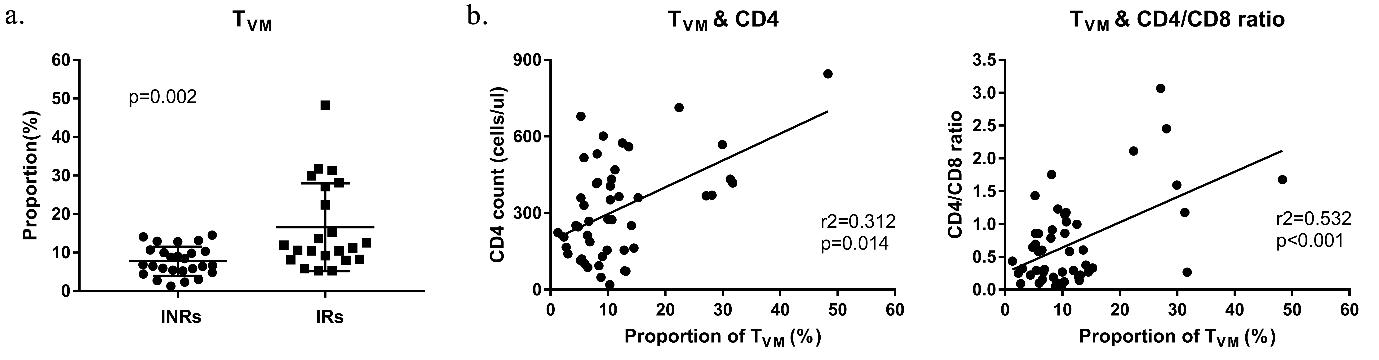


**Figure S6: HIV-1 DNA and multiple chemokines/cytokines after Tα1 treatment in cohort study.** (a) HIV-1 DNA. (b) IL-15. (c) CCL-3. (d) CCL-4. (e) CCL-5. Statistical method used in this figure was unpaired Student's t-test (n=3).


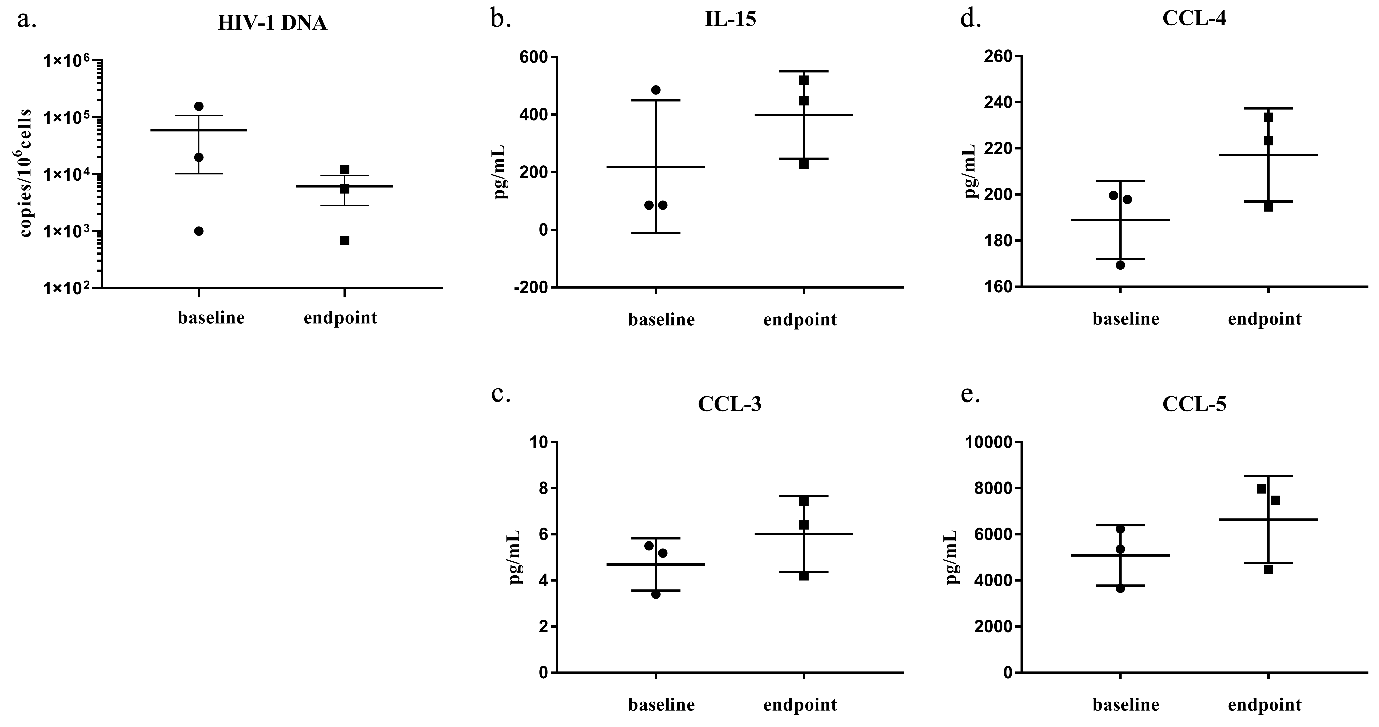

Supplement: Supplemental Material [file KVIR_A_2645858_SM2576.docx]
